# Novel Mechanistic Interplay between Products of Oxidative Stress and Components of the Complement System in AMD Pathogenesis

**DOI:** 10.4236/ojoph.2016.61006

**Published:** 2016-02-26

**Authors:** Hongjun Du, Xu Xiao, Travis Stiles, Christopher Douglas, Daisy Ho, Peter X. Shaw

**Affiliations:** 1 Department of Ophthalmology, Xijing Hospital, Xi’an, China; 2 Department of Ophthalmology and Shiley Eye Institute, University of California San Diego, San Diego, USA; 3 Sichuan Provincial People’s Hospital, Chengdu, China

**Keywords:** Age-Related Macular Degeneration, Oxidative Stress, Complement Factor H, Inflammation

## Abstract

Age-related macular degeneration (AMD) is a leading cause of vision loss affecting tens of millions of elderly worldwide. Early AMD includes soft drusen and pigmentary changes in the retinal pigment epithelium (RPE). As people age, such soft confluent drusen can progress into two forms of advanced AMD, geographic atrophy (GA, or dry AMD) or choroidal neovascularization (CNV, or wet AMD) and result in the loss of central vision. The exact mechanism for developing early AMD and progressing to advanced stage of disease is still largely unknown. However, significant evidence exists demonstrating a complex interplay of genetic and environmental factors as the cause of AMD progression. Together, complement factor H (CFH) and HTRA1/ARMS polymorphisms contribute to more than 50% of the genetic risk for AMD. Environmentally, oxidative stress from activities such as smoking has also demonstrated a powerful contribution to AMD progression. To extend our previous finding that genetic polymorphisms in CFH results in OxPLs and the risk-form of CFH (CFH Y402H) has reduced affinity for oxidized phospholipids, and subsequent diminished capacity which subsequently diminishes the capability to attenuate the inflammatory effects of these molecules, we compared the binding properties of CFH and CFH related protein 1 (CFHR1), which is also associated with disease risk, to OxPLs and their effects on modulating inflammation and lipids uptake. As both CFH-402H and CFHR1 are associated with increased risk to AMD, we hypothesized that like CFH-402H, CFHR1 contribution to AMD risk may also be due to its diminished affinity for OxPLs. Interestingly, we found that association of CFHR1 with OxPLs was not statistically different than CFH. However, binding of CFHR1 did not elicit the same protective benefits as CFH in that both inflammation and lipid uptake are unaffected by CFHR1 association with OxPLs. These findings demonstrate a novel and interesting complexity to the potential interplay between the complement system and oxidative stress byproducts, such as OxPLs, in the mechanistic contribution to AMD. Future work will aim to identify the molecular distinctions between CFH and CFHR1 which confer protection by the former, but not latter molecules. Understanding the molecular domains necessary for protection could provide interventional insights in the generation of novel therapeutics for AMD and other diseases associated with oxidative stress.

## 1. Introduction

It is established that AMD is a disease of aging and chronic inflammation. The hallmark of early AMD is lipid accumulation leading to the presence of drusen. Drusen are pockets of lipid and other extracellular material that aggregate between the Bruch’s membrane and the retinal pigment epithelium (RPE) of the eye [1]. Typically, sparse “hard” drusens are benign artifacts of aging but as the number and size (often leading to a “soft” designation) of drusen increase, so does the risk of progression to both forms of advanced AMD. The two forms of advanced AMD are: 1) geographic atrophy (GA) of the retinal pigment epithelium (RPE) and overlying photoreceptors (also called advanced “dry” AMD) and 2) choroidal neovascularization (CNV, also called “wet” AMD). GA AMD is characterized by confluent areas of photoreceptor and RPE cell death, and GA without CNV is responsible for over 10% of the legal blindness from advanced AMD [2]. Interestingly, just over half of GA AMD presents bilaterally, further demonstrating an environmental component of disease [3]. Currently, approximately 900,000 persons in the United States are affected by GA AMD [4]. However, despite the high correlation demonstrated between various genetic and environmental contributors the etiology remains largely unknown and there exists no approved treatment [5].

There is substantial evidence suggesting that contributing mechanisms to GA AMD require a combination of insults such as photo-oxidative stress, complement activation, cellular senescence, and microbial assault. This paradigm is supported by earlier work which demonstrated that loci at the complement factor H (CFH), C2 and C3 are associated with all phenotypic variants of AMD, including early AMD and GA [6]. Genes involved in lipid metabolism, such as ApoE and LIPC are also significantly associated with dry AMD [7] [8]. Central to many of these proposed factors, oxidative stress can have multifactorial influences amongst a number of the proposed pathways and is known to play a critical role in many aging diseases including cardiovascular disease and AMD [9]. Oxidative stress in the eye is of particular importance as the burden of purposeful sunlight exposure in combination with high metabolic demand and oxygen content creates a disproportionate burden of oxidative stress that requires tight homeostatic regulation to maintain function. A major product of this increased oxidative burden of the eye is a relatively large amount of OxPLs shed by photoreceptors [10]-[13]. These OxPLs are inherently inflammatory in systemic context, but the inflammatory burden is tightly controlled in the eye by largely unappreciated mechanisms. We hypothesize that, at least in part, functional abnormalities of the innate immune system incurred via high risk genotypes contribute to the pathogenesis of AMD by altering the tight homeostatic control of the OxPLs contribution to inflammation. This disruption leads to a state of chronic inflammation and pathologic progression.

The documented contribution of oxidative stress, inflammation, and genetic variations in complement factors indicates a strong probability that genetic risk modifies the aspects of the complement system in a manner which leads to perturbation and exacerbation of the cycle of oxidation, inflammation and pathogenic progression of this disease. We have shown that risk genotypes associated with CFH proteins interact with less affinity with OxPLs, and the decreased association leads to an increased inflammatory burden by these molecules [12]. The purpose of this study is to examine the potential mechanism underlying the increased risk of AMD progression that results from expression of CFHR1, and whether the contribution to the disease is reminiscent of phenomenon observed in the decreased interaction of CFH Y402H with OxPLs.

## 2. Material and Methods

### 2.1. Preparation of Native and Oxidized Phospholipid

Since phospholipids have low aqueous solubility, in plasma they typically co-exist with proteins in the form of lipoproteins, such as ApoB in low density lipoprotein (LDL). We thus chose LDL as a carrier for native or oxidized phospholipids moiety in this study. We first isolated LDL from plasma of normolipidemic donors (normal lipid level) by sequential ultracentrifugation [14]. OxLDL was generated by incubating LDL (1 mg/mL) with an oxidation agent (10 μM CuSO_4_) for 18 hours at 37°C where native phospholipids on the surface of LDL were oxidized into OxPLs [15]. Malondialdehyde-modified LDL (MDA-LDL), another form of oxidatively modified LDL, is made as described previously [16]. Native (non-oxidized) phospholipids moiety on native LDL were used as a control.

### 2.2. Preparation of Recombinant CFH and CFHR1

The gene encoding human CFH and CFHR1 was sub-cloned into a mammalian expression vector pRK5 [12]. The same amount of constructs was then transfected into HEK293 cells using Fugene 6 kit following the manufacturer’s instruction (Roche, Indianapolis, IN). The medium containing rCFH or rCFHR1 protein was harvested and concentrated using Amicon Ultra (Millipore, Billerica, MA). All recombinant proteins are assayed for endotoxin contamination using Gen Script ToxinSensor^TM^ Chromogenic-LAL Endotoxin Assay Kit to ensure the free of LPS and avoid the bias of the stimulation.

### 2.3. Chemiluminescent ELISA

For ELISA studies, lipoprotein antigens (in PBS, 2 μg/ml of protein concentration) were coated on microtiter plates at 4 C overnight. A 1:100 dilution of conditioned medium with the indicated construct was added to the plate, followed by biotinylated anti-CFH antibody (ab112197, abcam, Cambridge, MA, USA) or anti-CFHR1 antibody (ABIN1405357, antibodies-online, Atlanta, GA, USA). The amount of bound CFH or CFHR1 was detected with neutral avidin-alkaline phosphatase (AP) followed by light emission substrate Lumiphos 530. The chemiluminescence was measured and expressed as RLU/100 ms.

### 2.4. Culturing of RPE Cells and Stimulation

Human ARPE-19 cells were grown for 14 days in DMEM/F12 (1:1) plus 10% FCS to allow for forming of a truly differentiated epithelial layer. After serum starvation, cells were stimulated with oxidatively modified LDL or a control of non-modified LDL at 50 μg/ml for 18 hours (under our culture condition, most of inflammatory cytokine’s transcripts are exponentially expressed within 6 hours and most of gene transcripts peaked at 18 hours). To study the effect of binding of CFH or CFHR1 protein to OxLDL on stimulation of pro-inflammatory cytokines, we pre-incubated 50 μg/ml of OxLDL with concentrated conditioned media containing rCFH or rCFHR1 protein (50 μg/ml protein concentration by Thermo Scientific BCA assay). The OxPLs/CFH or CFHR1 complex was added to ARPE-19 cells for 18 hours incubation for gene expression or 48 hours for lipids accumulation.

### 2.5. Gene Expression Assays

After stimulation, RNA was purified using RNeasy Mini Kit (Qiagen) and used for cDNA synthesis using Superscript III (Invitrogen, Carlsbad, CA, USA). The expression of inflammatory cytokines/chemokines known to associated with AMD pathology was then assessed by quantitative PCR with SYBR Green Real-Time PCR Master Mixes (Thermo Fisher Scientific, Waltham, MA USA) using athermocycler (Bio-Rad Labs, Irvine, CA, USA). Sample sizes of 6 were used to reach the 80% of power when 0.25 fold change was expected, sigma = 0.25 and *α* = 0.05.

### 2.6. Western Blot

Cell extractions for protein assessment studies were performed as previously described [12]. Briefly, cells were lysed with Cell Lysis Buffer (#9803, Cell Signaling Technology, Boston, MA, USA) containing 0.5 mM of phenylmethanesulfonyl fluoride (PMSF) (Sigma-Aldrich, St. Louis, MO, USA). Protein concentration was standardized by BCA protein assay (Thermo Fisher Scientific, Grand Island, NY, USA). Samples (25 μg) were separated by SDS-PAGE in 4% – 20% gradient Tris-glycine precast gels (Invitrogen, Carlsbad, CA, USA) and transferred to a polyvinylidene difluoride (PVDF) membrane (Millipore, Billerica, MA, USA). The membrane was incubated for 1 hour in blocking solution containing 5% non-fat milk powder and 0.1% Tween-20, pH 7.6. This was followed by overnight incubation at 4 C in the blocking buffer containing rabbit primary antibodies against CD36 (Abcam, ab133625, 1:500). Subsequently, the labeled proteins were visualized by incubation with a horseradish peroxidase (HRP)-conjugated anti-goat or rabbit IgG (1:2000; Santa Cruz Biotechnology) followed by development with a chemiluminescence substrate for HRP (Thermo Fisher Scientific). The images of western blots were captured by GE imageQuant imager. Relative band intensities were analyzed using Image J software and normalized to GAPDH.

### 2.7. Oil-Red O Staining

Lipid accumulation in ARPE-19 cells was assessed *in vitro* as previously described [17]. Briefly, ARPE-19 cells were resuspended into 0.5 ml of DMEM containing 20% FCS and seeded to each well of a 12-well culture plate laid with a round cover slip. The following day, 25 μg/ml of native or OxLDL (50 μg/ml of OxLDL is toxic to cells during an extended incubation) as OxPLs/rCFH or rCFHR1 was added to the medium and extended the incubation for another 48 hours. After 48 hour of incubation, the cells were used to assess lipids uptake. The cells were washed with PBS and fixed with formaldehyde/sucrose solution and then stained with heated Oil red O/propylene glycol solution and mounted. The number of lipid droplets/cell of a total of 100 cells was counted using a microscope with a visual grid.

## 3. Results

### 3.1. Complement Factor H Related Protein 1 Has the Similar Binding Property to OxPLs as the Protective Form of CFH

**Figure 1:**
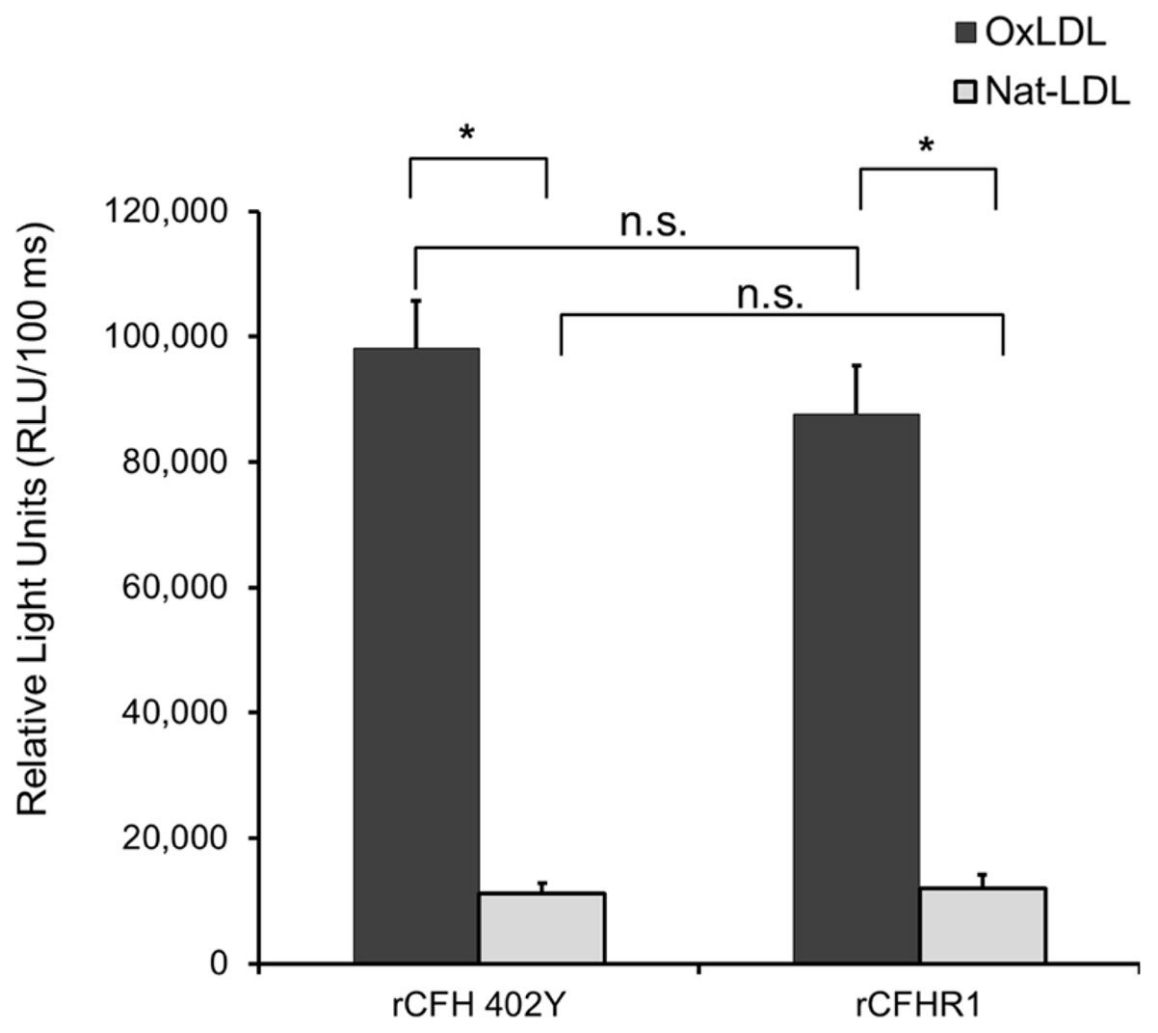
Relative binding property of CFH variants to oxidized phospholipids. The binding of recombinant protein rCFH 402Y (protective) and rCFHR1 to native or oxidized LDL antigens was measured using relative light units (RLUs) of chemiluminescence. Data are expressed in RLU and as mean ± SEM. N = 6 for each sample point, n.s., not significant, ^*^P < 0.05.

We previously showed that CFH derived from the risk (C) and protective (T) alleles bind differentially to OxPLs. Plasma containing CFH (rs1061170) homozygous CC (402H, risk genotype) vs. homozygous TT (402Y, protective genotype) in our experiment can be respectively exemplified with recombinant CFH402Y and CFH402H proteins, and as a result it can be shown that the protective form of CFH402Y binds to OxPLs stronger than risk form of CFH402H [12]. In this report, we also show that under the same assay conditions, both recombinant CFH and CFHR1 bind significantly stronger to OxPLs than to native LDL. However, there is no difference in binding to either native or OxLDL between CFH402Y and CFHR1 (Figure 1).

### 3.2. Oxidized Phospholipids Stimulate the Elevated Expression of Inflammatory Cytokines in Cultured RPE Cells

**Figure 2:**
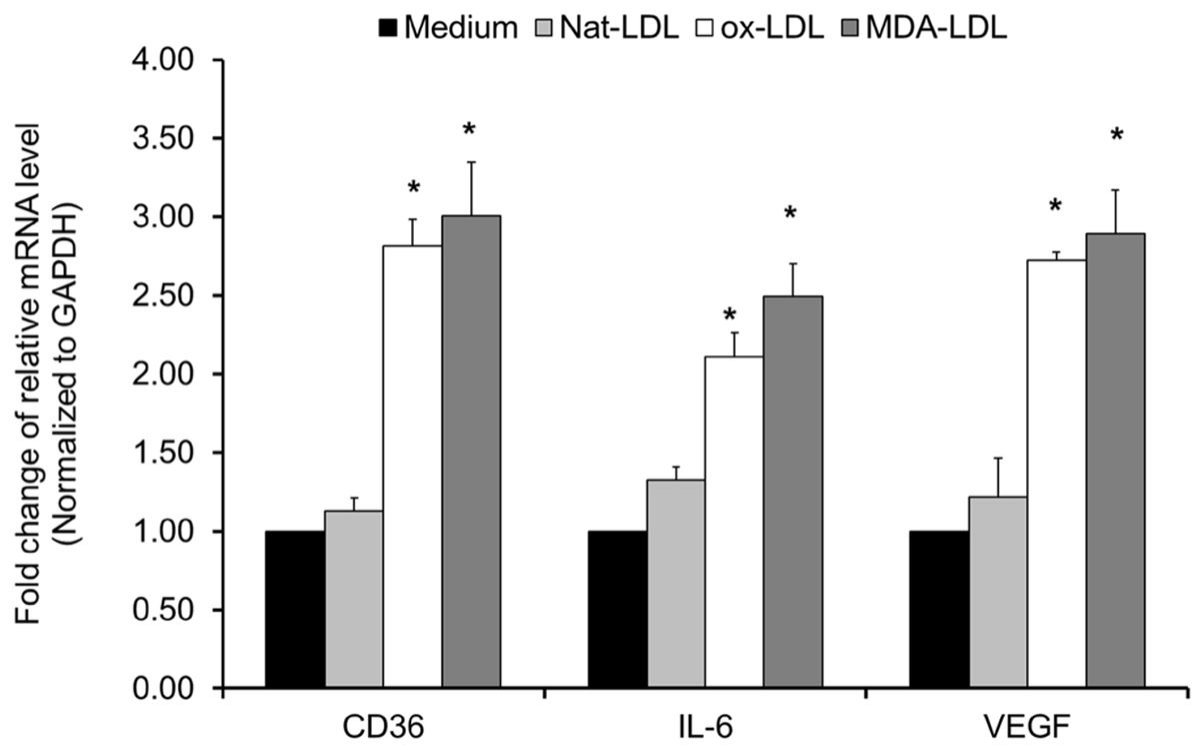
Gene expression as assayed by quantitative PCR on mRNA from ARPE-19 cells treated with 50 μg/mL of native LDL, OxLDL or MDA-LDL for 18 hour. Relative mRNA levels of indicated genes were calculated by normalizing results with GAPDH and are expressed relative to untreated samples. Data are shown as mean ± SEM. N = 6, ^*^P < 0.05.

RPE plays an important role in maintaining the health of the retina through outer segment lipid turnover [18]. Like macrophages, they engulf the released outer segment vesicles from photoreceptors. When extensive oxidative stress occurs, such as smoking and sunlight exposure, the RPE vesicles accumulate oxidative modified products like OxPLs, which in turn stimulate inflammation and the expression of cell-surface receptors, including CD36. CD36 is a scavenger receptor that will mediate uptake of OxLDL in an unregulated fashion [19]. These processes can lead to intracellular accumulation of debris and lipofuscin and further induction of cytokines critical for activation of pro-inflammatory cascades. We investigated gene-expression profiles in cultured ARPE-19 cells upon exposure to oxidative modified lipoproteins including OxLDL and MDA-LDL. Our results showed that comparing to native LDL control, the OxLDL and MDA-LDL significantly up-regulated the expression of scavenger receptor CD36, pro-inflammatory cytokine IL-6 and pro-angiogenic protein VEGF (Figure 2).

### 3.3. CFH and CFHR1 Protein Modulate CD36 Expression and Inflammatory Cytokine IL-6 Expression Stimulated OxPLs

**Figure 3:**
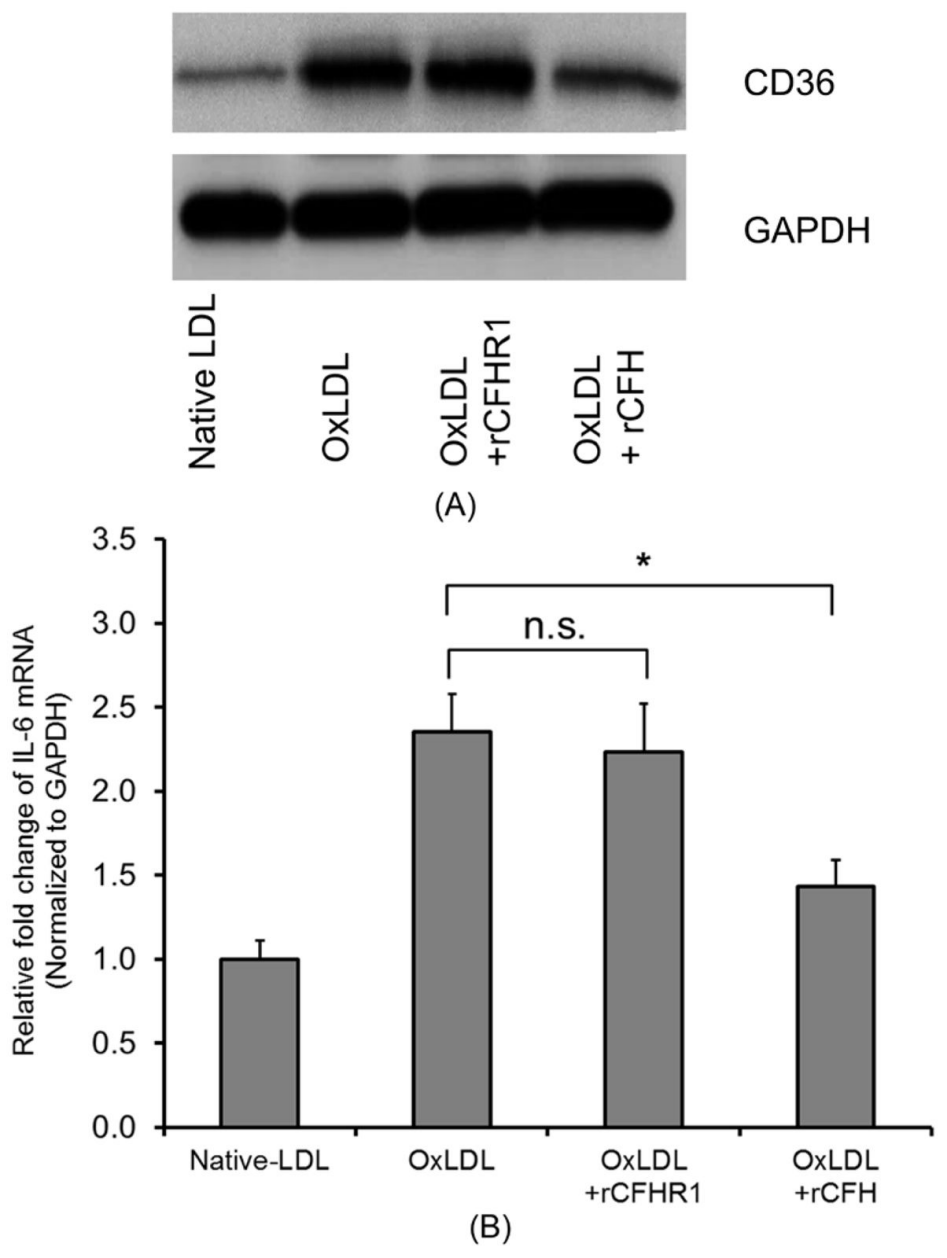
(A) CD36 protein level increased following treatment with OxLDL. ARPE-19 cells were treated with 50 μg/mL of native LDL, OxLDL or OxLDL and pre-incubated with the equal amount (50 μg/mL) of recombined rCFHR1 or rCFH as indicated, for 18 hours. The protein levels were assessed via SDS-PAGE and western blot using anti CD36 antibody. (B) CFH, but not CFHR1, inhibited expression of IL-6 stimulated by OxLDL. Gene expression was assayed by quantitative PCR on mRNA from ARPE19 cells treated with 50 μg/mL of native LDL, OxLDL or OxLDL pre-incubated with the equal amount (50 μg/mL) of recombined rCFHR1 or rCFH for 18 hours. Relative mRNA levels of indicated genes were calculated by normalizing results with GAPDH and are expressed relative to untreated samples. Results are shown as mean ± SEM. N = 6, ^*^P < 0.05.

To investigate whether the binding of CFH or CFHR1 would attenuate the scavenger receptor expression on ARPE-19 cells stimulated by OxPLs, we pre-incubated OxPLs with rCFH or rCFHR1. As expected, the interaction of rCFH with OxPLs significantly reduced the level of CD36 protein. Conversely, rCFHR1 failed to modulate the CD36 levels (Figure 3(A)) indicating an inability to recapitulate the protective benefits of CFH. The absence of protective capacity was further observed in assessing the IL-6 inflammatory response of ARPE-19 cells to OxPLs stimulation, which was significantly reduced by pre-incubation with rCFH but not with rCFHR1 (Figure 3(B)). These data indicated that even with the similar binding property to OxPLs, these two related proteins displayed different function in attenuating pro-inflammatory events *in vitro*.

### 3.4. Incubation of OxLDL Results in Lipids Accumulation in RPE Cells *in Vitro*

**Figure 4:**
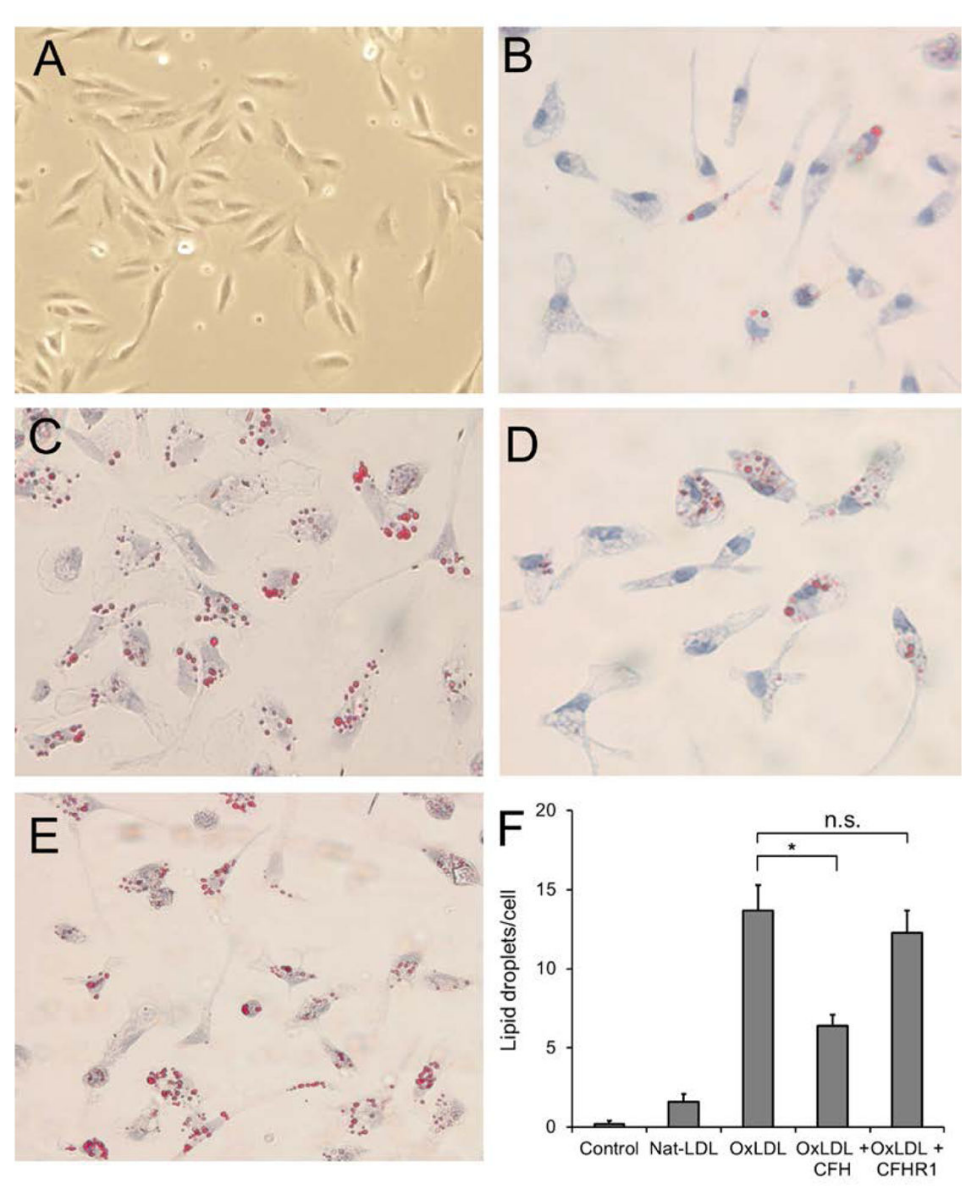
OxLDL3. Oxidized LDL enhances the uptake of lipids in cultured RPE cells. ARPE-19 cells were seeded and incubated for 48 hours with 25 μg/ml native-LDL (B), or 25 μg/ml OxLDL (C) alone, or OxLDL plus 25 μg/ml of rCFH (D), or OxLDL plus 25 μg/ml of rCFH (E). At the end of incubation, the cells were fixed and stained with Oil Red O. (A) is a picture of untreated ARPE-19 cells. (F), The lipid droplets were qualified for positive Oil Red O staining. Results are shown as stained lipid droplets/cell (mean ± SEM, ^*^P < 0.05).

As lipid-mediated inflammatory changes are closely linked to lipid accumulation and drusen formation in AMD, we extended the inflammatory characterization and investigated the lipids accumulation in cultured RPE cells following the *in vitro* incubation with native or OxLDL. To accomplish this, low-density seeded ARPE-19 (as shown in Figure 4(A)) were treated with 25 μg/ml concentration of native or OxLDL for 48 hours and assessed for lipid accumulation as visualized by Oil Red-O staining. As anticipated, the lipid vesicles were greatly increased in OxLDL treated RPE cells (Figure 4(C)) relative to native LDL treated cells (Figure 4(B)). Interestingly, addition of excess (50 μg/ml) of rCFH into the OxLDL containing medium led to significant reduction in the accumulation of lipids (Figure 4(D)), while the addition of rCFHR1 showed no significant effect on lipid accumulation (Figure 4(E)). Figure 4(F) shows the quantitative analysis of lipid droplets among untreated cells or cells treated with indicated agents. These data suggest that the scavenger receptor mediated uptake of OxLDL can be interfered by the binding of CFH to the OxPLs on the surface of OxLDL presumably by restricting the interaction between OxPLs and scavenger receptors. However, the interaction of CFHR1, which has the similar binding property as CFH, does not affect the OxLDL internalization.

## 4. Discussion

While we have uncovered increasingly more details concerning the speculative contributors to AMD progression, the mechanisms underlying how these factors intertwine to lead to advanced disease has largely remained elusive. Our previous work characterized a novel interaction of CFH with OxPLs, as well as mechanistic insight into how CFH proteins might restrict the inflammatory effects of these molecules. Additionally, we found that CFH variants associated with increased risk of AMD demonstrated a decreased affinity of the CFH protein for OxPLs, which led to a greater inflammatory burden [12]. The diminished protective capacity of these risk-associated variants in our studies represent a key mechanistic insight into the potential interplay between genetic risk factors and environmental stressors that cumulatively contribute to AMD progression. As a largely homologous protein to CFH, the fact that CFHR1 expression leads to increased AMD disease risk as opposed to protection has persisted as a somewhat enigmatic proposition. For example, while CFHR1 expression confers risk for AMD, it is protective against atypical hemolytic uremic syndrome [20]. As risk variants of CFH conferred disease risk via diminished association with OxPLs, we hypothesized that CFHR1 conferred risk by having similarly reduced OxPLs affinity, thereby enabling OxPLs to freely interact with inflammation-promoting factors. While CFHR1 is known to not be protective, we showed statistically identical association of CFHR1 and CFH with OxPLs. Indeed, the work represented herein demonstrates that OxPL/CFHR1 complexes offer no ability to attenuate the inflammatory effects of OxPLs. In addition to its ability to restrict inflammation in RPE cells, we had previously shown that the protective benefits of CFH extended to a diminished intake and accumulation of undesirable OxPL molecules, which logically would extend into a reduction in drusen formation. Despite its similar affinity and homology to CFH, CFHR1 does not impede the intake and accumulation of OxPLs into RPE cells, further shining light on its lack of protective capacity.

While the initial hypothesis of diminished OxPL association did not prove accurate, the work herein clearly demonstrates an intricate mechanism by which differential components of the CFH family contribute to AMD progression. What we conclude from this work is that simple association of CFH family members with OxPLs is not sufficient for blocking interactions between these molecules and RPE receptors which internalize these oxidized lipids. As this was a closed system with no other cellular contributors, the likely explanation for the observed phenomenon is that steric differences in the larger CFH protein somehow restrict, likely in a steric fashion, interaction of the OxPL molecules with RPE receptors that typically trigger internalization and inflammation. This paper indicates one of the most important aspects of complement regulators such as CFH and CFHR1. In addition to their protective function for complement activation triggered by external pathogens, CFH also blocks the inflammation triggered by OxLDL while CFHR1, loses such ability. Future work will focus on the molecular dissection of the CFH protein to elucidate the domains responsible for this restricted interaction in hopes of identifying the minimal essential components needed for the restriction of inflammation and lipid accumulation. By better understanding these mechanisms, we can provide valuable insight into both AMD pathology, and other conditions associated with oxidative stress. This will provide specific intervention points by which we can theorize and design future therapeutic approaches to improving outcomes of patients with these conditions.
